# Diagnostic Utility of Peripheral Blood *MYD88* L265P Testing in Vitreoretinal Lymphoma

**DOI:** 10.3390/genes17101172

**Published:** 2026-09-23

**Authors:** Almila Sarigul Sezenoz, Aslan Aykut, Rajesh C. Rao, Victor M. Elner, F. Yesim Demirci, Noah Brown, Hakan Demirci

**Affiliations:** 1 Kellogg Eye Center, Department of Ophthalmology and Visual Sciences, University of Michigan, Ann Arbor, MI 48105, USA; almilasarigul@gmail.com (A.S.S.); aslanaykut81@gmail.com (A.A.); rajeshr@med.umich.edu (R.C.R.); velner@med.umich.edu (V.M.E.); 2 Department of Ophthalmology, Baskent University, Ankara 06490, Turkey; 3 Department of Ophthalmology, Marmara University, Istanbul 34899, Turkey; 4 Kellogg Eye Center, Department of Pathology, University of Michigan, Ann Arbor, MI 48105, USA; 5 Department of Human Genetics, School of Public Health, University of Pittsburgh, Pittsburgh, PA 15261, USA; fyd1@pitt.edu; 6 Department of Pathology and Clinical Labs, University of Michigan, Ann Arbor, MI 48105, USA; noahbr@med.umich.edu

**Keywords:** intraocular fluid, molecular diagnostics, *MYD88* L265P, peripheral blood, vitreoretinal lymphoma

## Abstract

Background/Objectives: Vitreoretinal lymphoma (VRL) is a rare form of non-Hodgkin lymphoma. Molecular analysis of *MYD88* mutations in intraocular fluids (aqueous or vitreous humor) has emerged as a valuable diagnostic tool in VRL. The purpose of this study was to evaluate the diagnostic utility of peripheral blood *MYD88* L265P testing in VRL and its relationship with central nervous system (CNS) involvement. Methods: Patients presenting with suspected VRL between 03/2021 and 03/2024 were included. Aqueous humor samples were collected from all patients during the initial office visit. Peripheral blood samples were obtained from all patients before treatment. All patients were evaluated for the presence of the *MYD88* (NM_002468.4) c.794T>C (p.L265P) mutation in their aqueous humor and peripheral blood samples by using an allele-specific, real-time polymerase chain reaction assay. VRL diagnosis was established clinically and confirmed by vitreous cytopathology. Vitreous humor was also tested for *MYD88* L265P mutation when vitrectomy was done at our institution. Results: Twelve patients (eight males, four females) with VRL were included. Seven patients had CNS involvement, and four were on systemic chemotherapy at the time of sampling. Aqueous humor-derived *MYD88* L265P mutation was identified in 9/12 patients (75.0%), and vitreous humor-derived *MYD88* L265P mutation was identified in 8/9 patients tested (88.9%). The mutation was not identified in any of the blood samples. Conclusions: While aqueous and vitreous humor samples demonstrated high detection rates for the *MYD88* L265P mutation in VRL, allele-specific real-time PCR analysis of predominantly leukocyte-derived DNA extracted from the whole blood did not detect the *MYD88* L265P mutation. This finding supports the concept that VRL, including cases with CNS involvement, appears to be mainly confined to immune-privileged sites with no detectable circulating lymphoma cells for useful peripheral blood DNA analysis.

## 1. Introduction

Vitreoretinal lymphoma (VRL) is the most common form of lymphoma in the eye and a rare subtype of non-Hodgkin lymphoma. It may present as primary VRL or in association with central nervous system lymphoma (CNSL). VRL and CNSL are closely linked as 50–90% of patients with primary VRL have concurrent or subsequent CNSL, while 15–25% of patients with primary CNSL develop VRL [1,2]. In this study, the term VRL includes both the cases that initially presented in the eye and the cases associated with CNSL.

Myeloid differentiation primary response gene 88 (*MYD88*) L265P mutation is one of the most frequent mutations detected in primary CNSL (60% of cases) and primary VRL (87% of cases) [3,4]. Our previous studies have demonstrated the diagnostic and disease monitoring utility of *MYD88* mutation analysis of aqueous and vitreous humor liquid biopsies obtained from patients with primary VRL [2,5]. In addition, *MYD88* mutations have been detected in cerebrospinal fluid (CSF) cell-free DNA (cfDNA) of primary VRL patients even without clinically apparent CNS involvement [1].

Recent studies have shown that plasma circulating tumor DNA (ctDNA) analysis can provide a minimally invasive approach to detect *MYD88* mutations in primary CNSL patients, reflecting the presence of tumor-derived DNA fragments in the circulation [6,7,8,9]. On the other hand, analysis of cellular DNA extracted from peripheral blood leucocytes would address a different biological question, as to whether mutation-bearing lymphoma cells are present in peripheral circulation. The *MYD88* testing used in our laboratory was primarily developed for the detection of lymphoplasmacytic lymphoma, in which the mutation would be detected in circulating lymphocytes in peripheral blood specimens. Therefore, detection of *MYD88* mutations in peripheral blood leukocytes of patients with VRL would suggest the presence of circulating lymphoma cells and/or systemic disease rather than lymphoma confined to immune-privileged sites.

Given the increasing interest in minimally invasive molecular testing, we conducted this study to evaluate whether peripheral blood *MYD88* L265P testing, using a routine clinical assay performed on predominantly leukocyte-derived (cellular) DNA extracted from whole blood, has diagnostic value in patients with VRL and/or in those with CNS involvement. Our analyses did not include plasma or serum cfDNA testing as the blood-related validated cfDNA extraction/processing protocols were not available at the time of our study. Therefore, our findings focus on predominantly cellular DNA obtained from whole blood and do not reflect the diagnostic performance of blood-based cfDNA analysis.

## 2. Materials and Methods

This study was carried out prospectively at Kellogg Eye Center, University of Michigan, with approval of the institutional review board, and it adhered to the tenets of the Declaration of Helsinki.

Patients who presented with suspected VRL at Kellogg Eye Center, University of Michigan, between March 2021 and March 2024 were included in the study. Informed consent was obtained from each patient. Information was collected on demographics, involved eye, laterality, and CNS involvement. Patients underwent evaluation for the *MYD88* L265P mutation in aqueous humor, vitreous humor (when available), and blood samples at Molecular Diagnostics Laboratory of University of Michigan. Patients with a previous history of Waldenström macroglobulinemia and/or IgM monoclonal gammopathy of undetermined significance were excluded from the study.

All patients were consulted with the neuro-oncology department for the evaluation of any extraocular and CNS involvement of the disease.

### 2.1. Aqueous and Vitreous Humor Collection

While patients with concurrent CNS or systemic lymphoma were diagnosed in the context of their known lymphoma, in patients who presented without CNS or systemic involvement, the diagnosis of VRL was established by cytopathologic analysis of vitreous samples. If the vitrectomy was performed at our institution, *MYD88* L265P mutation analysis of the vitreous sample was also conducted. For vitreous sampling, at the beginning of surgery, approximately 1–1.5 mL of undiluted vitreous was collected prior to initiation of infusion. At the conclusion of the vitrectomy procedure, the diluted vitreous collected in the vitrectomy cassette was also submitted for diagnostic evaluation. Both vitreous samples were evaluated as part of our routine diagnostic protocol for VRL. Aqueous humor samples were collected during the initial ophthalmology visit at the office from all patients. Approximately 0.2 mL aqueous humor was drawn from the anterior chamber with a 30-gauge needle through the temporal limbus and the specimen was sent immediately for genetic testing.

### 2.2. DNA Isolation and MYD88 Testing of Ocular Fluids

The vitreous and/or aqueous humor obtained from each patient was centrifuged and cellular and cell-free DNA (cfDNA) were extracted using the Quick-DNA urine kit (Zymo Research, Irvine, CA, USA), as previously described [5]. Due to the low and variable DNA yields, a standard fixed DNA input could not be used, and all available extracted DNA was included in the analysis. All samples were evaluated for the *MYD88* (NM_002468.4) c.794T>C (p.L265P) mutation at Molecular Diagnostics Laboratory of University of Michigan by an allele-specific, real-time polymerase chain reaction assay. This assay specifically targeted the *MYD88* L265P mutation, and other *MYD88* variants were not evaluated by using sequencing methods. A control amplification reaction was conducted simultaneously to verify the integrity of the DNA sample. The detection threshold for this clinical assay was established at a 1% mutant allele frequency.

### 2.3. Blood Sample Collection and Processing

Peripheral blood samples were obtained from all patients before administration of any ocular treatment. Samples were collected in EDTA and subjected to an automated whole blood DNA extraction procedure using the BioRobot EZ1 Advanced system (Qiagen, Hilden, Germany). Predominantly leukocyte-derived (cellular) DNAs extracted from the whole blood samples were then tested for the *MYD88* L265P mutation by allele-specific, real-time PCR, as described above. A standard DNA input of 25 ng was used for analysis.

### 2.4. Assay Validation and Sample Adequacy

The limit of detection of this *MYD88* assay was empirically validated for fresh specimens, including blood, and formalin-fixed paraffin-embedded (FFPE) tissues. For fresh specimens, cell line DNA was diluted with DNA extracted from the peripheral blood of healthy individuals to achieve variant allele frequencies of 40%, 10%, 5%, 1%, 0.5%, 0.1% and 0.01%. A similar experiment was performed for paraffin-embedded tissue by sequential dilution of DNA from an FFPE block from a known positive case, in which the variant allele frequency was determined to be 40% by ancillary testing, in which the diluent was FFPE normal bone marrow aspirate clot section. The mutation was detected, based on the delta cycle threshold (Ct) cutoff for this assay, down to a variant allele frequency of 0.5% for both fresh and FFPE specimens. Based on these findings, the limit of detection was conservatively set at 1.0%.

Separate dilution studies were not performed for ocular fluids because of the low volumes and DNA yields from such samples. During validation, a 1% FFPE control was evaluated 7 times on separate runs and yielded a positive result in all runs. Clinical cases in the validation (not in the dilution studies) included 30 peripheral blood samples expected to be negative, all of which were tested negative, as well as 1 peripheral blood and 5 fresh bone marrow aspirates from patients diagnosed with lymphoplasmacytic lymphoma, all of which were found to be positive.

To establish a criterion for adequacy, the 0.5% mutation-positive FFPE sample, representing the lowest-quality DNA sample type in the dilution study, was serially diluted with water to obtain progressively lower DNA inputs. The 0.5% mutation was consistently detected when the control Ct was ≤30 cycles; therefore, a control Ct of 30 cycles was defined as the threshold for adequate results. The *MYD88* L265P mutation analysis was considered inadequate when the Ct value for the wild-type control reaction exceeded 30 cycles. In these samples, the stated limit of detection could not be reliably reached, and the results therefore could not be regarded as definitively negative. Finally, a 1% positive control is also included in every run of this clinical assay and has yielded a positive result in every run since the assay was validated 10 years ago.

### 2.5. Statistical Analysis

Categorical variables were summarized as numbers and percentages. The 95% confidence intervals (CIs) for the detection rates of the *MYD88* mutation were calculated using the Wilson method. Comparative analysis for *MYD88* mutation positivity between patients with and without CNS involvement was performed by using Fisher’s exact test. A *p* value < 0.05 was considered statistically significant.

## 3. Results

A total of 20 eyes of 12 patients (eight males, four females) were included in this study. The median age of the patients was 70.5 years (range: 55–85 years). At the time of analysis, eight patients (66.7%) had bilateral involvement, while four of them (33.3%) had unilateral disease. The diagnosis of VRL was confirmed cytopathologically in all patients. Seven patients (58.3%) had a history of CNS involvement. Five of them (41.7% of the overall cohort) had concurrent active CNS disease at the time of VRL diagnosis, and four of them were on systemic chemotherapy at the time of data collection. Cerebrospinal fluid samples were not analyzed for the *MYD88* L265P mutation as part of this study. One patient (Patient 7) had a history of cutaneous lymphoma (diffuse large B-cell), treated 7 years prior; however, he had no systemic involvement at the time of VRL diagnosis. Another patient (Patient 11) had a history of breast lymphoma treated systemically 20 years prior. At the time of ocular sampling, all affected eyes had vitreous cells, and 11 also had retinal involvement. Sixteen eyes had no anterior segment involvement while three had diffuse keratic precipitates, and one had 3+ anterior chamber cells (Table 1).

### 3.1. Detection of MYD88 L265P Mutation in Intraocular Fluid Samples

Aqueous and/or vitreous humor samples showed positivity for the *MYD88* L265P mutation in nine of 12 patients (75.0%; 95% CI, 46.8–91.1%) in total (Table 1). Aqueous humor-derived *MYD88* L265P mutation was detected in nine of 12 patients tested (75.0%; 95% CI, 46.8–91.1%). Vitreous biopsy was performed in 12 eyes of nine patients as the remaining eight eyes had vitrectomy elsewhere. The vitreous humor-derived *MYD88* L265P mutation was detected in eight of nine patients tested (88.9%; 95% CI, 56.5–98.0%).

Of 20 aqueous humor samples (from 12 patients), six samples (from five patients) were classified as inadequate. Four of these patients had an adequate aqueous humor result from the fellow eye, which was positive in three patients and negative in one. One patient had inadequate aqueous humor results in both eyes and no vitreous sample available. All vitreous humor samples yielded adequate results. When the analysis was restricted to patients with at least one adequate aqueous humor result, *MYD88* L265P was detected in nine of 11 patients tested (81.8%; 95% CI, 52.3–94.9%).

Among the three patients in whom *MYD88* L265P was not detected using intraocular fluid samples, one had a clinical diagnosis suggestive of VRL, while two had primary CNSL with ocular involvement. Among the seven patients with current or a history of CNS involvement, five (71.4%; 95% CI, 35.9–91.8%) had detectable *MYD88* L265P in intraocular fluid samples, one had a negative test result, and one had an inadequate aqueous humor result without any vitreous humor sample available, despite positive clinical VRL findings. Among patients with adequate intraocular fluid results, *MYD88* L265P was detected in five of six patients with CNS involvement and four of five patients without CNS involvement (83.3% vs. 80.0%, respectively; Fisher’s exact test, *p* = 1.000). However, given the small subgroup sizes, this comparison should be considered exploratory.

In 12 eyes, both aqueous and vitreous samples were available for analysis, while in three of them, the analysis showed inadequate results. After excluding the inadequate results, all nine eyes showed concordant results in aqueous and vitreous humor analyses (100%; 95% CI, 70.1–100%). Both aqueous and vitreous samples were tested positive in eight eyes and negative in one eye.

### 3.2. Detection of MYD88 L265P Mutation in Peripheral Blood

The *MYD88* L265P mutation was not detected in any of the 12 whole blood samples analyzed (0%; 95% CI, 0–24.3%). All peripheral blood samples yielded adequate results. Detailed information on each patient can be found in Table 1.

## 4. Discussion

In this study, we investigated the *MYD88* L265P mutation positivity in peripheral blood samples from VRL patients to evaluate its potential as a diagnostic tool in VRL overall and in patients with CNS involvement. Despite a significant rate of mutation being detected in aqueous and vitreous humor samples (75.0% and 88.9%, respectively), no mutation was found using real-time PCR analysis of the predominantly leukocyte-derived (cellular) DNAs extracted from peripheral blood samples. Our results suggest that while *MYD88* L265P is a valuable biomarker in intraocular fluid samples, its detection in peripheral blood samples remains challenging, especially when based on routine whole blood DNA extraction and analysis.

Previous studies have reported *MYD88* L265P mutation rates of 67–75% in aqueous humor and 75–89% in vitreous humor samples, with a high concordance between paired vitreous and aqueous samples in VRL patients [2,5,10,11]. Our findings align with the literature showing a high positivity of *MYD88* L265P mutation in intraocular fluid samples. Similarly, we detected a 75.0% positivity in aqueous and 88.9% positivity in vitreous samples, with 100% concordance among the paired samples with adequate results, by using real-time allele-specific PCR. Our findings further underscore the diagnostic value of intraocular liquid biopsies in VRL patients. In addition to cellular or circulating cell-free DNA, nucleic acids carried by extracellular vesicles, including miRNAs, are being studied as potential biomarkers in ocular biofluids and may offer another approach to molecular profiling in ocular diseases [12,13].

To our knowledge, our study is the first to investigate the *MYD88* L265P mutation in predominantly leukocyte-derived (cellular) DNAs extracted from whole blood samples of patients with VRL, while no studies are currently available on plasma- or serum-derived cfDNA analysis in these patients. On the other hand, CSF-derived cfDNA has previously been studied in patients with VRL [1], and both CSF- and plasma-derived cfDNAs have previously been evaluated as potential diagnostic tools in patients with primary CNSL [6,7,8,9,14]. It was shown that the concentration of plasma cfDNA in primary CNSL patients was lower compared to the patients with systemic lymphomas [6]. While there is no consensus yet, Mutter et al. [14] were able to detect ctDNA in 78% of plasma samples in a cohort of 92 PCNSL patients while Yoon et al. [8] observed ctDNA in only 27% of 41 patients. The reported detection rates of *MYD88* mutations in blood-derived cfDNA from patients with primary CNSL have ranged from 0% to 57%, depending on the analytical method and whether serum or plasma was analyzed [6,7,8,9,14]. Yoon et al. [8] have shown the *MYD88* mutation in plasma ctDNA of three out of 41 (7%) primary CNSL patients, while Hiemcke-Jiwa et al. [9] detected the *MYD88* L265P mutation in two out of 10 (20%) primary CNSL patients by evaluating their plasma samples. In CSF-derived cfDNA, the *MYD88* L265P mutation was found in 73% of samples obtained from primary CNSL patients [9]. Moreover, Zhuang et al. [1] reported that the *MYD88* L265P mutation was observed in the CSF of VRL patients with a 63.2% sensitivity. *MYD88* L265P is not only a useful diagnostic biomarker but was also shown to promote the NF-κB and JAK–STAT3 signaling pathways that support malignant B-cell survival, thus highlighting some potential therapeutic targets [15].

In our study, despite the high diagnostic yield of intraocular fluid samples (by using both cell-free and cellular DNA analysis), the *MYD88* L265P mutation was undetectable in any of the peripheral blood samples of our VRL patients (by using automated whole blood extraction, thus mainly leukocyte-based cellular DNA analysis), including the patients with concurrent CNS lymphoma. Our findings of 0% peripheral blood positivity indicate that *MYD88* L265P is not detectable by testing predominantly leukocyte-derived (cellular) DNA with the current assay sensitivity (with a 1% variant allele frequency detection threshold). Taken together with the high detection rate in intraocular fluids, our findings support the compartmentalized biology of VRL and CNSL, suggesting that mutation-carrying lymphoma cells may have limited access to peripheral circulation. This observation is consistent with VRL and CNSL arising and remaining localized within immune-privileged compartments, potentially limiting the detectability of tumor-derived material in peripheral blood with the standard methods used. However, our results should be interpreted with caution, as the low-frequency variants (<1%) may not be detectable with this approach. Therefore, our results cannot distinguish between a true absence of circulating mutation-bearing cells and the presence of these cells at levels below the 1% detection limit of the assay used.

Importantly, a negative *MYD88* L265P whole blood test would not exclude VRL. Of note, this study did not employ the isolation of cfDNA from plasma or serum, and the standard whole blood procedures are not expected to efficiently capture cfDNA. Therefore, absence of detectable *MYD88* mutations in peripheral blood using mainly leukocyte-derived (cellular) DNA likely reflects further limited sensitivity of this approach, as it mainly relies on tumor-derived DNA from lymphoma cells entering the bloodstream, rather than the cfDNA released into the blood by these cells. Hence, while predominantly leukocyte-derived (cellular) DNA testing depends mainly on the presence of mutation-bearing cells in the circulation, plasma/serum cfDNA analysis may detect low levels of tumor-derived DNA fragments released into the blood even in the absence of circulating lymphoma cells. Accordingly, our findings do not exclude the potential diagnostic value of plasma or serum cfDNA analysis in VRL patients using more sensitive methods.

Despite being the first to analyze the peripheral blood in VRL, our study had several limitations. First, as stated above, *MYD88* L265P testing in peripheral blood was performed on predominantly leukocyte-derived (cellular) DNA rather than plasma-/serum-derived cfDNA, as blood-related cfDNA extraction/processing protocols were not available at the time of our study. Therefore, our results do not address the potential diagnostic value of plasma/serum cfDNA analysis using higher-sensitivity methods. Likewise, our study did not include CSF-derived cfDNA analysis, which limited our ability to compare our blood findings with CSF findings. Our findings are limited to routine *MYD88* mutation testing of predominantly leukocyte-derived (cellular) DNA using whole blood, as performed in standard clinical molecular diagnostics laboratories, reflecting real-world practice. Secondly, the sample size was limited, which is inherent to the rarity of VRL, and the cross-sectional nature of our study did not allow for longitudinal assessment of peripheral blood testing. Finally, our study did not include CD19-positive B-cell enrichment before DNA analysis because of our purpose to evaluate routine whole blood testing as used in clinical practice. Previously, it was shown that CD19-positive B-cell sorting can improve the detection of the *MYD88* L265P mutation in Waldenström macroglobulinemia [16]. Therefore, as also stated above, it is possible that rare circulating B-cells carrying the mutation may have remained below the detection limit in our study.

## 5. Conclusions

Our results have important implications for the management of patients with VRL. Our findings further support that intraocular liquid biopsy investigating the *MYD88* L265P mutation acts as a sensitive diagnostic tool. On the other hand, our data show that a negative peripheral blood test based on the analysis of mainly leukocyte-derived cellular DNA (from routine whole blood processing) using an assay with a 1% variant allele frequency detection threshold should not be interpreted as definite evidence for the absence of disease. These findings are specific to the testing approach we used and do not address plasma/serum cfDNA analysis or more sensitive methods such as digital PCR or next-generation sequencing approaches. Therefore, clinicians should continue to prioritize intraocular fluid testing and, when clinically indicated, CSF testing for the *MYD88* L265P mutation as a diagnostic tool in patients suspected to have VRL without or with CNS involvement.

As this is the first study to investigate peripheral blood positivity in VRL patients, our findings contribute valuable data to the growing body of research but also indicate the need for further investigation by employing plasma/serum cfDNA analysis, more sensitive methods, and/or longitudinal monitoring in larger patient cohorts, in order to advance our knowledge of the potential utility of minimally invasive blood-based mutation analysis in VRL.

## Figures and Tables

**Table 1:** The demographic characteristics, clinical findings, and mutation analyses results of the study patients.

Patient	Age	Sex	Involved Eye	Anterior Segment Involvement	Vitreous and Retinal Involvement	*MYD88* L265P Mutation in Aqueous Humor	*MYD88* L265P Mutation in Vitreous Humor	*MYD88* L265P Mutation in Leukocyte-Based Blood Cellular DNA	CNS Involvement
1	71	Male	Right	+3/4 cells	+3/4 cells	Positive	N/A	Negative	Positive
			Left	No cells	2+ cells	Positive	Positive		
2	83	Male	Right	No cells, but diffuse KP	1+ cells and retinal involvement	Positive	Positive	Negative	Positive
3	73	Male	Right	No	1+ cells and retinal involvement	Positive	N/A	Negative	Negative
			Left	No	1+ cells and hazy view	Inadequate	Positive		
4	70	Male	Right	No	Vitreous opacities	Positive	Positive	Negative	Negative
			Left	No	Vitreous opacities	Positive	Positive		
5	68	Male	Right	No	2+ cells and retinal involvement	Negative	N/A	Negative	Positive
6	74	Female	Right	No	Trace cells, no retinal involvement	Positive	Positive	Negative	Negative
			Left	No	Trace cells no retinal involvement	Inadequate	Positive		
7	76	Male	Right	No cells, KPs	1+ cells and retinal involvement	Inadequate	Negative	Negative	Negative
			Left	No cells, KPs	1+ cells and retinal involvement	Negative	N/A		
8	77	Female	Right	No	1+ cells no retinal involvement	Positive	Positive	Negative	Negative
			Left	No	1+ cells, no retinal involvement	Negative	Negative		
9	55	Male	Right	No	1+ cells	Inadequate	N/A	Negative	Positive
			Left	No	1+ cells	Inadequate	N/A		
10	69	Male	Left	No	Trace cells and retinal involvement	Positive	Positive	Negative	Positive
11	62	Female	Right	No	2+ cells and retinal involvement	Positive	Positive	Negative	Positive
12	59	Female	Right	No	2+ cells and retinal involvement	Positive	N/A	Negative	Positive
			Left	No	Trace cells and retinal involvement	Inadequate	N/A		

CNS: central nervous system, N/A: not applicable, KP: keratic precipitate, Inadequate: Results were considered inadequate when the cycle threshold for the wild-type reaction was above the assay cutoff. In these samples, the stated limit of detection could not be reliably reached, and the results therefore could not be regarded as definitively negative.

## Data Availability

All data supporting the findings of this study are available within the paper.

## Abbreviations

The following abbreviations are used in this manuscript:

VRL	Vitreoretinal lymphoma
CNS	Central nervous system
CNSL	Central nervous system lymphoma
*MYD88*	Myeloid differentiation primary response gene 88
PCR	Polymerase chain reaction
DNA	Deoxyribonucleic acid
cfDNA	Cell-free DNA
ctDNA	Circulating tumor DNA
CSF	Cerebrospinal fluid
